# Effects of Sol–Gel Modification on the Microstructure of Nafion Membranes

**DOI:** 10.3390/polym17111542

**Published:** 2025-06-01

**Authors:** Ruslan M. Mensharapov, Nataliya A. Ivanova, Artem V. Bakirov, Anna S. Semkina, Timofey D. Patsaev, Matvey V. Sinyakov, Olga I. Klein, Petr V. Dmitryakov, Caizhi Zhang, Dmitry D. Spasov

**Affiliations:** 1 National Research Center “Kurchatov Institute”, 1, Akademika Kurchatova sq., 123182 Moscow, Russia; mensharapov_rm@nrcki.ru (R.M.M.); bakirov_av@nrcki.ru (A.V.B.); semkina_as@nrcki.ru (A.S.S.); timpatsaev@mail.ru (T.D.P.); sinyakov_mv@nrcki.ru (M.V.S.); klein_oi@nrcki.ru (O.I.K.); dmitryakov_pv@nrcki.ru (P.V.D.); 2 “D.V. Sokolsky Institute of Fuel, Catalysis and Electrochemistry” JSC, 142 Kunayev Str., Almaty 050010, Kazakhstan; ivanovana.1989@mail.ru; 3 Enikolopov Institute of Synthetic Polymeric Materials, Russian Academy of Sciences, 117393 Moscow, Russia; 4 Department of Isotope Technology and Hydrogen Energy, Institute of Modern Energetics and Nanotechnology, D. Mendeleev University of Chemical Technology of Russia, Miusskaya sq., 9, 125047 Moscow, Russia; 5 Bach Institute of Biochemistry, Research Center of Biotechnology of the Russian Academy of Sciences, 119071 Moscow, Russia; 6 College of Mechanical and Vehicle Engineering, Chongqing University, Chongqing 400044, China; czzhang@cqu.edu.cn; 7 National Research University “Moscow Power Engineering Institute”, 14, Krasnokazarmennaya St., 111250 Moscow, Russia

**Keywords:** proton exchange membrane, Nafion, sol–gel method, silica nanoparticles, small-angle X-ray scattering, ionic domains

## Abstract

Proton exchange membrane (PEM) is a key component of PEM fuel cells, where the membrane plays a decisive role in determining system efficiency and overall performance. The modification of PEMs with hydrophilic dopants represents a promising strategy for extending the operational range of these devices, particularly in low-humidity and high-temperature regimes. In this study, Nafion membranes were modified with silica nanoparticles via the sol–gel method; samples with 1, 3, 5, and 10 wt.% of SiO_2_ were obtained. Evaluation of key parameters demonstrated improvement of water uptake and proton conductivity for modified membranes with silica content up to 5 wt.%, while no significant changes in thermal stability (30–700 °C) were observed. The structural changes in the composite membranes were investigated using the small-angle X-ray scattering (SAXS) technique. SAXS data were analyzed using a model-dependent approach: the spherical ionic domain model was modified to account for the scattering contribution from silica nanoparticles. The results obtained demonstrated a reduction in the size of unmodified ionic domains, indicating reorganization of the composite membrane’s microstructure.

## 1. Introduction

With the increasing role of hydrogen technologies in the global energy sector, enhancing the performance and durability of proton exchange membrane fuel cells (PEMFCs) has become a critical challenge [1]. However, the widespread adoption of PEMFCs in power systems is hindered by their strong dependence on operating conditions, particularly relative humidity and ambient temperature [2,3,4,5,6,7]. The parameters of the proton exchange membrane (PEM), which transports protons and separates reagents, are critical for PEMFC performance. At elevated temperatures (>60–80 °C) and low humidity (<40–60%), PEMFCs may exhibit reduced efficiency or even failure due to PEM dehydration, as their ionic conductivity is highly dependent on the level of water sorption [8,9]. Furthermore, the membrane’s properties influence the structural stability and integrity of PEMFC components under sub-zero conditions, since ice formation on the PEM surface is governed by the amount of water desorbed from the membrane during freezing [10,11].

**Figure 1:**
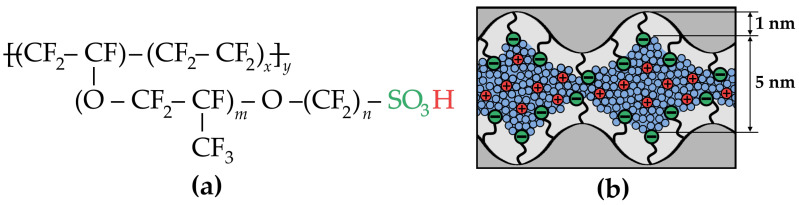
(**a**) Chemical structure of the PFSA ionomer (Nafion-type membrane). (**b**) Schematic illustration of the hydrated ionic domain structure in the PEM. Sulfonic groups are shown in green, protons in red, water in blue, and polymer chains in gray and black.

Under standard PEMFC operating conditions, perfluorinated sulfonic acid (PFSA) membranes exhibit optimal stability and conductivity. These membranes, represented by the general chemical formula in Figure 1a, include commercial variants such as Nafion^®^ (DuPont, Wilmington, DE, USA; *m* = 1, *n* = 2, *x* = 5–7), Aquivion^®^ (Solvay, Brussels, Belgium; *m* = 0, *n* = 2, *x* = 6–7), and 3M PFSA^®^ (3M, Saint Paul, MN, USA; *m* = 0, *n* = 4, *x* = 3–6) [12,13]. Their chemical stability and mechanical strength arise from hydrophobic perfluorinated backbones with robust C–F and C–C bonds. Meanwhile, flexible side chains terminating in hydrophilic sulfonic acid groups enable self-organization into hydrated ionic domains (Figure 1b), ensuring high proton conductivity [9,14]. The size of the formed ionic domains is dependent on the water content, reaching approximately 5 nm for a fully hydrated membrane (20–25 wt.% H_2_O). Beyond affecting the morphology of these domains, water molecules play a dual role in proton conduction by facilitating both vehicular (transport) and Grotthuss mechanisms [15,16]. Consequently, optimization of water content becomes a critical parameter when adapting PEM properties for extreme PEMFC operating conditions.

Inorganic hydrophilic additives, such as silica nanoparticles and metal oxides (TiO_2_, ZrO_2_, Al_2_O_3_, etc.), provide a cost-effective solution for stabilizing the water balance in PEMs [17,18,19,20]. Nafion^®^ composite membranes demonstrate 10–40% greater water retention capacity compared to pristine membranes under elevated temperatures (80–120 °C) across a broad humidity range [21,22,23]. This enhanced water retention improves proton conductivity by 10–50% under demanding operating conditions (30% relative humidity, 80 °C) [24,25]. Doping the membrane with 5 wt.% SiO_2_ reduced the rate of PEMFC power output degradation by 20-fold at 100 °C and 20% gas humidity [26]. The hydrophilic surface oxygen groups of the SiO_2_ nanoparticles enhance water retention, improving membrane stability at sub-zero temperatures. This effect is attributed to suppressed water desorption during freezing, which mitigates degradation of the PEMFC catalytic layers [27].

PEM modification can be carried out by the following two main approaches: (1) membrane formation from a prepared ionomer suspension and modifier nanoparticles and (2) impregnation of the fabricated membrane in a mixture containing prepared dopant nanoparticles or their precursors, followed by in situ formation of modifying particles in the PEM matrix [28,29,30]. The first method of manufacturing modified PEM is characterized by relative simplicity and short manufacturing time and allows direct control over the size, structure, and concentration of the modifier. There are also no restrictions on the type of dopant used, since its fabrication is carried out independently and the incorporation is performed by mixing with a suspension of ionomer and solvent followed by homogenization [31]. The main disadvantage of this method is the disruption of the initial macromolecular structure of the perfluorinated membrane framework, the reduction in crystallinity of the polymer matrix, as well as the agglomeration and precipitation of dopant particles during drying of the suspension, which leads to a decrease in the mechanical stability of PEM and non-uniform distribution of the modifier [18,32].

The in situ PEM modification method comprises three main stages: swelling, impregnation, and drying [33]. The first stage involves membrane immersion in a solvent (methanol, ethanol, or dimethyl sulfoxide) to induce membrane expansion and increase ionic domain size [34,35]. The subsequent impregnation stage introduces modifier nanoparticles or their precursors into the PEM. When using pre-synthesized nanoparticles, they penetrate the PEM volume through diffusion, uniformly distribute through the channels, and become anchored in the polymer matrix via hydrogen bonding [32]. For controlled synthesis of nanoparticles with specific size and structure, the sol–gel method proves particularly effective. This method typically employs alkoxide precursors, particularly tetraethyl orthosilicate (TEOS), for SiO_2_ synthesis. The precursor diffuses into the PEM channel network where, in the presence of water and under the catalytic action of sulfonic groups, sequential hydrolysis and condensation reactions occur, leading to the formation of small branched modifier nanoclusters [36,37,38]. Since the diffusion efficiency of either precursors or pre-formed modifier nanoparticles depends on the average size of the swollen membrane’s ionic channels, the dopant size generally does not exceed 7 nm, while the modifier concentration is typically limited to a maximum of 3–5 wt.% [18]. The in situ sol–gel method is consistent with existing membrane manufacturing methods, but introducing additional stages increases the complexity of composite membrane production [9].

For comprehensive PEM studies, including modifier content optimization and analysis of its effect on water distribution in ionic domains, small-angle X-ray scattering (SAXS) serves as a universal approach alongside traditional physicochemical methods. The characteristic peak in SAXS *I*(*q*) curves corresponding to PEM ionic domain scattering (the ionomer peak) typically appears in the 1 < *q* < 2 nm^−^¹ range [39,40]. Analysis of this peak employs model-dependent approaches based on theoretical concepts of domain geometry. The most frequently used models include spherical domains [41,42,43], cylindrical channels [44,45], and locally lamellar clusters [44,46]. The accuracy of membrane structure description using these idealized models varies with PEM hydration level [47]. Proper interpretation of the ionomer peak requires consideration beyond the form factor, including (1) local ordering of adjacent ionic domains through structure factor introduction, (2) electron density contrast between the polymer matrix and ionomer side chains, and (3) size distribution polydispersity [43,44,48]. For SAXS analysis of modified PEMs, the modifier contribution is typically neglected, with experimental curve processing employing approaches identical to those for standard membranes [49,50].

In this study, bulk modification of Nafion^®^ 212 membranes was performed using the in situ sol–gel method, producing samples with SiO_2_ content ranging from 1 to 10 wt.%. The fundamental physicochemical parameters of the composite PEMs were obtained, revealing a positive contribution of modification to overall water uptake and conductivity for samples containing up to 5 wt.% SiO_2_.

To investigate structural changes in the modified PEMs, SAXS patterns were acquired for water-hydrated membranes. An optimal model was selected to describe the experimental data of the pristine PEM and subsequently adapted to account for dopant nanoparticles localized within the ionic domains.

Using the obtained physicochemical and structural parameters of the composite PEMs, a model was proposed to describe the reorganization of the internal membrane structure under the influence of SiO_2_ nanoparticles.

## 2. Materials and Methods

### 2.1. Main Materials

Nafion^®^ 212 membranes were purchased from DuPont (Wilmington, DE, USA). Tetraethyl orthosilicate (TEOS, ≥ 99.5%) was purchased from EKOS-1 (Moscow, Russia), ethanol was obtained from Merck (Moscow, Russia), and nitric acid (HNO_3_) was obtained from Vekton (Saint Petersburg, Russia). All solutions and suspensions were prepared using DI water. All materials were used directly as supplied without additional purification.

### 2.2. Membrane Preparation

In this study, Nafion^®^ 212 membranes with a thickness of 50.8 microns and an equivalent weight of 1100 g·mol^−1^ were used. Prior to modification, the membranes were converted to protonated (H^+^) form. To remove surface contaminants, the membrane was treated in a 10% aqueous nitric acid solution at 95 °C for 1 h. The acid solution was then decanted, and the membrane was rinsed with DI water. Subsequently, the membrane was immersed in DI water at 95 °C for 1 h with two water changes until neutral pH was achieved. Prepared membranes were stored in DI water prior to modification. For dry weight determination, membranes were vacuum-dried in an oven at 110 °C for 1 h before modification.

Bulk PEM modification was performed via in situ sol–gel reaction. Pretreated membranes were first immersed in 60 vol.% aqueous ethanol solution at room temperature for 2 h to expand membrane channels and enhance reagent diffusion. The samples were then rinsed with ethanol to remove surface water and transferred to a mixture of 200 mL TEOS and 100 mL ethanol. In this aqueous–alcoholic environment, the sol–gel process occurred within the PEM channels under the catalytic action of sulfonic groups, resulting in SiO_2_ nanoparticle formation. The synthesis was conducted at room temperature, with SiO_2_ concentration controlled by varying the reaction time between 1 and 5 min. The upper reaction time limit was determined by the absence of further sample weight gain. The modified membranes were thoroughly washed with ethanol to remove residual precursor and unstable surface particles. Membranes were vacuum-dried at 110 °C for 1 h to completely remove solvent and promote final SiO_2_ formation from residual TEOS within the PEM matrix. Additional protonation was achieved by repeating the nitric acid treatment following the aforementioned protocol. Modifier concentration in the final samples was calculated based on initial and final dry weights.

### 2.3. Membrane Characterization

Water uptake. The prepared membrane samples were completely dried in a vacuum oven at 110 °C, and the dry sample mass (*m_dry_*) was measured. Subsequently, the membranes were immersed in DI water at room temperature for 24 h.

Prior to measuring the wet sample mass (*m_wet_*), excess surface moisture was carefully removed by blotting. The water uptake was then calculated according to Equation (1).

(1) $$w.u.=\frac{m_{wet}-m_{dry}}{m_{dry}},$$

Conductivity. Prior to conductivity measurements, PEM samples were equilibrated in DI water at room temperature until complete hydration was achieved. The through-plane proton conductivity was determined using a two-electrode cell configuration. Electrodes were connected to a CorrTest CS350 electrochemical workstation (CorrTest Instruments, Wuhan, China) equipped with impedance modules. Measurements were conducted in the frequency range of 0.1–10⁶ Hz with an AC amplitude of 20 mV. Experimental data were fitted using an equivalent circuit model (*R_m_*, *CPE_m_*)(*R_i_*, *CPE_i_*), where the parallel combination of membrane resistance (*R_m_*) and constant phase element (*CPE_m_*, *Z_CPE_ = [A*(*jω*)*^n^*]^−1^, *A* is the proportionality coefficient, *ω* is angular frequency [rad·s^−1^], *j* is the imaginary unit, and *n* represents phase deviation) describes the bulk membrane impedance, and the parallel interface resistance (*R_i_*) and CPE_i_ account for interfacial impedance effects arising from membrane surface roughness and electrode geometry [51]. The through-plane resistivity ($\rho_{m}$) and conductivity (*σₘ*) were calculated using the following equation:

(2) $$\rho_{m}=\frac{1}{\sigma_{m}}=\frac{R_{m}\cdot h\cdot \delta }{L},$$

where *h* is the membrane width (*h* = 15 mm), *δ* denotes the thickness (*δ* = 50 μm), and *L* represents the interelectrode distance (*L* = 20 mm).

Electron microscopy. The samples were examined using an Osiris scanning/transmission electron microscope (STEM) (Thermo Fisher Scientific, Waltham, MA, USA) equipped with a high-angle annular dark field (HAADF) detector (Fischione, Export, PA, USA) and a Super-X EDX spectrometer (Bruker, Billerica, MA, USA) for chemical composition analysis (equipment of the Center of Collective Use of the Federal Scientific Research Centre “Crystallography and Photonics” of the Russian Academy of Sciences, Moscow, Russia, was used). The images were obtained at an accelerating voltage of 200 kV. The exposure time was optimized to minimize electron beam-induced damage to the membrane material.

Silicon distribution analysis in SiO_2_-modified membranes was performed using a Helios Nanolab 600 dual-beam scanning electron microscope (SEM) (Thermo Fisher Scientific, Waltham, MA, USA) equipped with an EDAX (AMETEK, Berwyn, PA, USA) detector for energy-dispersive X-ray spectroscopy (EDS) and a secondary electron Everhardt-Thornley detector (ETD). Images were obtained at an accelerating voltage of 5 kV and a current of 11 nA. Prior to analysis, the membrane samples were embedded in Epon 812 epoxy resin, and their cross-sections were polished with aluminum oxide particles. To prevent charging effects, the analyzed surface was sputter-coated with a thin gold layer.

Thermogravimetric analysis. The SiO_2_ concentration in composite membrane samples was verified, and the thermal stability of the polymer matrix was evaluated by thermogravimetric analysis (TGA). Measurements were performed on dry samples using a Mettler Toledo TGA/DSC3+ simultaneous thermal analyzer in dynamic mode (equipment of the resource center «Polymer» of the National Research Center “Kurchatov Institute”). The temperature range was set from 30 to 700 °C under a nitrogen flow (99.999% purity) of 50 mL·min^−1^ with a heating rate of 10 °C·min^−1^. Standard open ceramic crucibles (75 μL volume) were used for all measurements. The temperature measurement accuracy was ±0.1 °C, while the mass measurement precision reached ±0.001 mg.

Small-angle X-ray scattering. The nanostructure of hydrated polymer electrolyte membranes was investigated using small-angle X-ray scattering (SAXS). This technique enables direct examination of the phase-separated polymer structure, particularly for determining the average size of ionic domains, without requiring sample pretreatment. SAXS patterns for both pristine and bulk-modified membranes were acquired using an S3-MICRO SAXS camera system (Hecus, Graz, Austria) equipped with a Xenocs Genix X-ray source (operating at 50 kV, 1 mA) generating CuKα radiation (*λ* = 1.542 Å) with a beam size of 950 × 250 μm × μm. A two-dimensional Pilatus 100K detector (DECTRIS, Baden-Dättwil, Switzerland) was employed for signal collection. To minimize air scattering effects, both the X-ray optics path and measurement chamber were evacuated to 2–3 × 10^−2^ mmHg. Fully hydrated membrane samples (equilibrated in deionized water) were measured under vacuum conditions. To prevent dehydration, samples were sandwiched between thin polyimide films and hermetically sealed. Background scattering contributions from the polyimide films were systematically subtracted by acquiring separate reference scans of blank films.

Reference SAXS measurements were additionally performed at the BioMUR beamline at the Kurchatov Synchrotron Radiation Source (Moscow, Russian Federation) [52]. The X-ray optical components featured a domestic upgrade of the HZG beamline (DESY, Hamburg, Zeuthen, Germany). A 2D Pilatus3 1M detector system (DECTRIS, Baden-Dättwil, Switzerland) was employed, enabling photon-counting detection with a resolution of 1043 × 981 pixels. The measurements were conducted at an X-ray energy of 8 keV (*λ* = 0.1445 nm) with a beam size of 500 × 350 μm × μm.

The acquired data consisted of two-dimensional scattering patterns, which were converted into one-dimensional scattering profiles (intensity versus scattering angle *θ* or scattering vector *q*, nm^−1^) through radial integration using the Fit2D software package [53]:

(3) $$q=\frac{4\cdot \pi \cdot \sin\left(\frac{\theta }{2}\right)}{\lambda }$$

The hydrated polymer electrolyte membrane exhibits a lamellar structure with an interlayer spacing of 10–20 nm and ionic domains of nanoscale dimensions (typically several nanometers). Accordingly, the SAXS profile displays two distinct peaks. Applying the method’s nominal resolution equation (*d* = 2*π*/*q*), the low-*q* peak (*q* ≈ 0.3–0.5 nm^−1^) corresponds to the polymer’s lamellar organization, while the high-*q* peak (*q* ≈ 1–2.5 nm^−1^, hydration-dependent) arises from ionic channels. To fully resolve these features, measurements were performed across a *q*-range of 0.02–6 nm^−1^.

**Figure 2:**
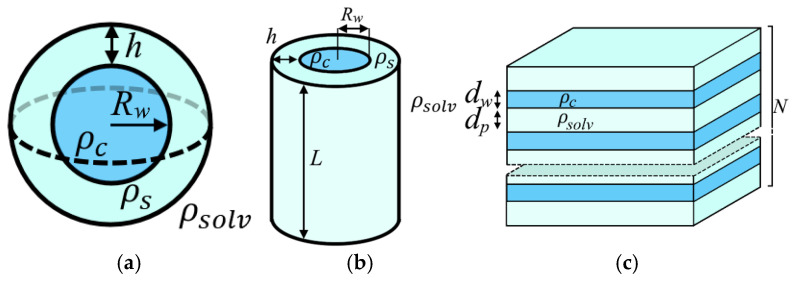
Schematic representation of (**a**) spherical water domains, (**b**) cylindrical channels, and (**c**) lamellar clusters.

The small-angle scattering profiles *I*(*q*) were fitted using theoretical models implemented in the SASfit software package (version 0.94.10) [54], with additional optimization performed via the curve_fit function from Python 3.x’s SciPy library. Three distinct structural models of hydrated polymer electrolyte membranes were evaluated: (1) a core–shell model with spherical domains [43], (2) cylindrical channel morphology [44], and (3) lamellar clusters [47]. Schematic representations of these models are presented in Figure 2.

According to the first model, the ionic domain consists of a spherical core with radius *R_w_* and a scattering length density corresponding to water (*ρ_c_* = 9.412·10^10^ cm^−2^). This central sphere is surrounded by a shell of thickness *h*, representing the polymer side chains and sulfonic groups that form the inner surface of ionic channels (*ρ_s_* = 16.147·10^10^ cm^−2^). The ionic domains are embedded in an amorphous polytetrafluoroethylene matrix (*ρ_solv_* = 15.443·10^10^ cm^−2^). The form factor for such spherical domains is given by the following equations:

(4) $$F\left(q\right)={\left(\rho_{c}-\rho_{s}\right)R}^{3}\Phi_{s}\left(qR_{w}\right)+{\left(\rho_{s}-\rho_{solv}\right)\left(R_{w}+h\right)}^{3}\Phi_{s}\left(q\left(R_{w}+h\right)\right)$$

(5) $$\Phi_{s}\left(qR_{w}\right)=3\frac{\sin qR_{w}-qRcos\;qR_{w}}{{\left(qR_{w}\right)}^{3}}$$

**Figure 3:**
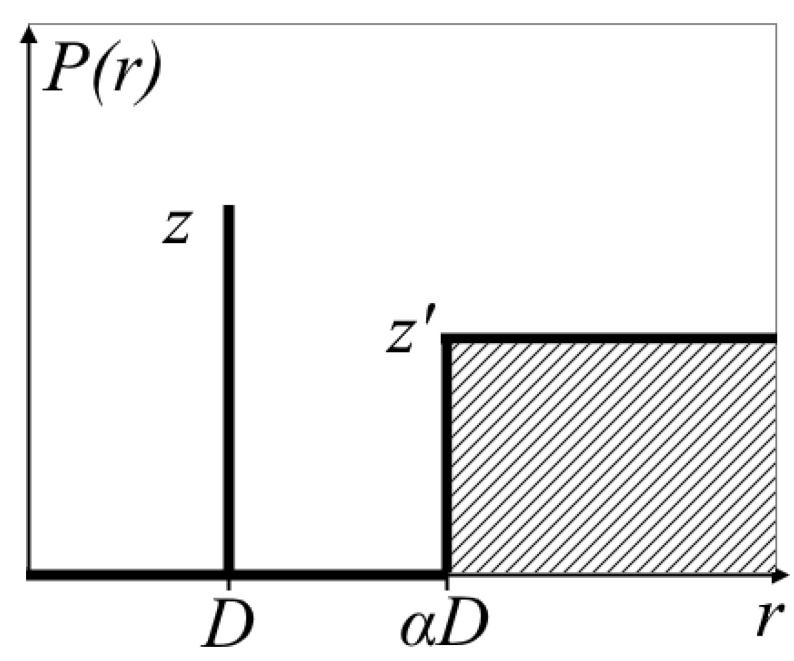
Radial distribution function of water clusters [48].

The structure factor accounts for the short-range ordering of domains, where each ionic domain is surrounded by four nearest neighbors at a distance *D*. A correlation hole exists between *D* and *αD* (where no other domains are present), while beyond *αD* the domains are randomly distributed [48]. The corresponding radial distribution function is shown in Figure 3.

Thus, the structure factor and the final intensity expression are given by the following equations:

(6) $$S\left(q\right)=1+\frac{1}{V}\int_{0}^{\infty }\frac{\sin qr}{qr}\left(P\left(r\right)-1\right)4\pi r^{2}dr=\;1+z\frac{\sin qD}{qD}-z\prime \frac{3(\sin\alpha qD-\alpha qD\cos\alpha qD)}{{\left(\alpha qD\right)}^{3}}$$

(7) $$\hat{I}\left(q\right)\;\sim \;S\left(q\right)*{\left(F\left(q\right)\right)}^{2}$$

The coordination number *z* = 4 corresponds to the number of neighboring clusters, while the effective coordination parameter *z′* ≈ 4 and the scaling factor α ≈ 1.1–1.2. The interdomain distance *D* is derived from the ratio of the water cluster volume to the volume of a cubic unit cell with edge length $2D/\sqrt{3}$, using the volume fraction of water in the hydrated membrane *φ_w_*:

(8) $$D={R_{w}\left(\frac{\sqrt{3}\pi }{2\varphi_{w}}\right)}^{1/3},\varphi_{w}=\frac{w.u.\times d_{Naf}}{w.u.\times d_{Naf}+d_{H_{2}O}}$$

where *d_Naf_* is the density of the Nafion^®^ membrane (2.1 g·cm^−3^), $d_{H_{2}O}$ is the density of water (1.0 g·cm^−3^), and *w.u.* denotes the water uptake of the membrane.

In the second model, water domains are represented as cylindrical channels, each comprising a water-filled core surrounded by a shell of sulfonic groups and polymer side chains. The channel length *L* is held constant, with parallel alignment assumed for neighboring channels. To improve agreement with experimental data, the theoretical fitting incorporated a structure factor accounting for hexagonal packing of these cylindrical channels. The radial distribution function for this arrangement is analogous to that shown in Figure 3. An analytical expression was derived for the structure factor. Consequently, the form factor (in cylindrical coordinates) and structure factor are given by the following equations:

(9) $$\Phi_{c}\left(qR_{w}\right)=2\frac{J_{1}\left(qR_{w}\right)}{qR_{w}}$$

(10) $$F\left(q\right)={\left(\rho_{p}-\rho_{s}\right)R_{w}}^{2}\Phi_{c}\left(qR_{w}\right)+{\left(\rho_{s}-\rho_{solv}\right)\left(R_{w}+h\right)}^{2}\Phi_{c}\left(q\left(R_{w}+h\right)\right)$$

(11) $$S\left(q\right)=1+\frac{1}{V}\int_{0}^{\infty }J_{0}(qr)\left(P\left(r\right)-1\right)2\pi Ldr=1+zJ_{0}(qD)-z\prime \frac{{2J}_{1}(\alpha qD)}{\alpha qD}$$

(12) $$D=R_{w}{\left(\frac{2\pi }{\sqrt{3}\varphi_{w}}\right)}^{1/2}$$

where *z*, *z′* ≈ 2, *α* ≈ 1.1–1.2, *J_0_*, and *J_1_* denote the Bessel functions of the first kind of order zero and one, respectively. For both models, size polydispersity for domain and channel sizes *R* was accounted for in the scattering intensity *I* through a normal distribution *f*(*R*) with mean value *R_w_* and standard deviation *R_w_σ_R_*:

(13) $$I\left(q\right)=\int_{0}^{\infty }f(R)\hat{I}\left(q,R\right)dR\;$$

In the lamellar cluster model, the membrane structure consists of alternating planar polymer and water layers with thicknesses *d_p_* and *d_w_*, respectively, and a periodicity *d = d_p_ + d_w_*. The model accounts for positional disorder between adjacent water layers, characterized by a root-mean-square fluctuation Δ in the interlayer spacing. Additionally, the contribution of non-structured layers (*N_diff_*) is incorporated. The form factor and structure factor for this lamellar cluster model are expressed as follows:

(14) $$\Phi_{l}\left(q\right)=\frac{\sin\left(qd_{w}/2\right)}{qd_{w}/2}$$

(15) $$F\left(q\right)=\left(\rho_{w}-\rho_{c}\right)d_{w}\Phi_{l}\left(q\right)$$

(16) $$S\left(q\right)=N_{diff}+\sum_{N_{k}=N-2\sigma }^{N+2\sigma }x_{k}(N_{k}+2\sum_{m=1}^{N_{k}-1}\left(N_{k}-m\right)\cos\left(mqd\right)exp(-\frac{mq^{2}{∆}^{2}}{2}))$$

(17) $$\sigma =\left\{\begin{array}{c} \sqrt{N},N\geq 5\\ \left(N-1\right)/2,\;N<5 \end{array}\right.\;,\;x_{k}=\frac{1}{\sigma \sqrt{\pi }}\exp\left(-\frac{{\left(N_{k}-N\right)}^{2}}{2\sigma^{2}}\right)$$

where *N* is the average number of water layers [46].

During SAXS data processing for the surface-modified sample, the scattering intensity from modifier particles was subtracted from the experimental curve prior to analysis, accounting for their high concentration in the surface layer and absence in the bulk polymer matrix.

Theoretical SAXS curves for bulk SiO_2_-modified polymer electrolyte membranes were simulated using a spherical domain model incorporating two distinct cluster types: modified and unmodified. The model assumes uniform spatial distribution of modified clusters throughout the membrane volume. The scattering intensity was calculated as follows:

(18) $$I=\nu_{mod}I_{mod}+(1-{\nu_{mod})I}_{stand}$$

where $\nu_{mod}$ is the volume fraction of modified clusters and $I_{stand}$ is the scattering intensity from unmodified clusters (given by Equation (7)). $I_{mod}$ is the scattering intensity from modified clusters, calculated as the square of Equation (3). The scattering length density was determined from the volume fraction of SiO_2_ ($\nu_{SiO_{2}}$) within the domain:

(19) $$\rho_{c}\prime ={\nu_{SiO_{2}}\rho_{SiO_{2}}+(1-\nu_{SiO_{2}})\rho }_{c}$$

(20) $$\nu_{SiO_{2}}=\frac{\varphi_{SiO_{2}}}{\left(\varphi_{SiO_{2}}+\frac{\varphi_{w}}{1-\varphi_{w}}\right)\nu_{mod}}$$

where $\rho_{SiO_{2}}$ is a scattering length density corresponding to SiO_2_ ($\rho_{SiO_{2}}$= 17.150·10^10^ cm^−2^) and $\varphi_{SiO_{2}}$ is the volume fraction of SiO_2_ in the PEM, which is calculated from its mass fraction ($w_{SiO_{2}}$) and the density of amorphous silica ($d_{SiO_{2}}$ = 2.0 g·cm^−3^) using the following equation:

(21) $$\varphi_{SiO_{2}}=\frac{{d_{Naf}w}_{SiO_{2}}}{w_{SiO_{2}}\left(d_{Naf}-d_{SiO_{2}}\right)+d_{SiO_{2}}}$$

To evaluate the agreement between theoretical (*I_theor_*) and experimental (*I_exp_*) scattering profiles *I*(*q*), we calculated the *R-factor*, defined as follows:

(22) $$R-\mathrm{factor}=\frac{//I_{exp}/-|I_{theor}|/}{/I_{exp}/}$$

## 3. Results and Discussion

### 3.1. Results of Membrane Modification

Through sol–gel synthesis, we prepared a series of 5 × 5 cm × cm samples with modifier concentrations ranging from 1 to 10 wt.%. It should be emphasized that the success of sol–gel modification critically depends on several factors: the presence of residual surface moisture on the PEM after soaking in water–alcohol solution, the mixing quality of the TEOS/ethanol solution, and the thoroughness of membrane surface rinsing to remove TEOS residues after synthesis. Insufficient removal of water from the membrane surface prior to synthesis, as well as excessive TEOS remaining on the PEM surface after synthesis, resulted in predominant formation of SiO_2_ particles on and near the membrane surface, along with the formation of large silica gel domains. This effect significantly distorts the determined bulk modifier concentration and increases membrane brittleness. Inadequate mixing of the TEOS/ethanol solution led to non-uniform SiO_2_ distribution, creating localized regions within the PEM with high modifier concentration and elevated fragility.

Based on the findings reported in [18], the maximum achievable bulk modifier content in PEMs modified by this method does not exceed 6 wt.%. For samples with higher nominal concentrations, we anticipate surface deposition of SiO_2_ particles. This study focuses on a series of membranes with modifier mass fractions of 1, 3, 5, and 10 wt.%.

### 3.2. Water Uptake

Water uptake and hydration number ($\lambda_{H_{2}O}$—water molecules per -SO_3_^−^ group) measurements at room temperature yielded values of 24 ± 1% ($\lambda_{H_{2}O}=14.7\;\pm 0.6)$, 25 ± 1% ($\lambda_{H_{2}O}=15.3\;\pm 0.6)$, 28 ± 2% ($\lambda_{H_{2}O}=17.1\;\pm 1.2)$, 31 ± 2% ($\lambda_{H_{2}O}=18.9\;\pm 1.2)$, and 33 ± 2% ($\lambda_{H_{2}O}=20.2\;\pm 1.2)$ for bulk-modified PEMs containing 0, 1, 3, 5, and 10 wt.% SiO_2_, respectively. This systematic increase in hydration capacity with modifier concentration directly correlates with the hydrophilic nature of SiO_2_. Notably, surface-modified samples exhibited enhanced water uptake due to the presence of large pores in the deposited layer, which facilitate greater water sorption capacity.

### 3.3. Conductivity

**Figure 4:**
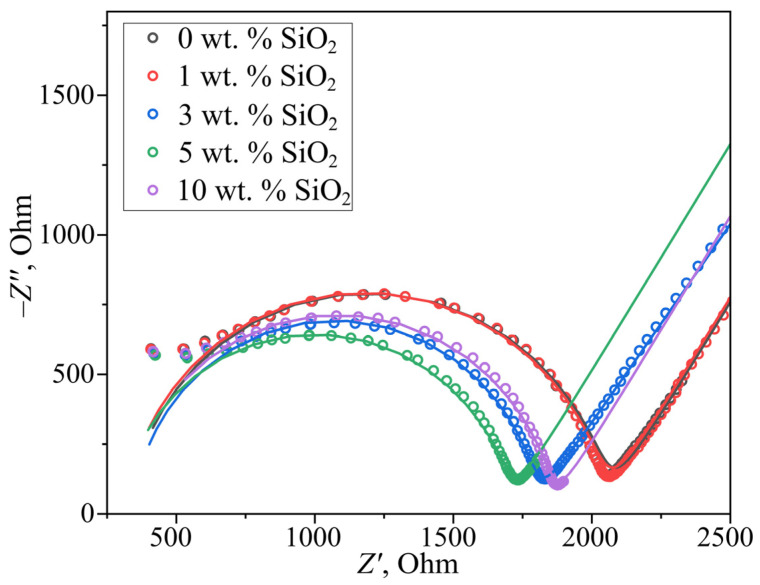
Nyquist plots of bulk-modified PEMs. The fitting results are displayed as solid lines.

The membrane conductivity was investigated by electrochemical impedance spectroscopy, with Nyquist plots shown in Figure 4. For bulk-modified PEMs, the conductivity values measured 0.156 ± 0.005, 0.156 ± 0.005, 0.185 ± 0.006, 0.192 ± 0.006, and 0.173 ± 0.005 S·cm^−1^ for samples containing 0, 1, 3, 5, and 10 wt.% modifier, respectively. The observed conductivity increase (up to 5 wt.% SiO_2_) correlates with both elevated water uptake and the constrained elasticity effect of aqueous domain walls described in [18]. This phenomenon involves dopant-induced water redistribution from ionic domains to interdomain channels, causing channel expansion and enhanced proton transport. Notably, when the SiO_2_ concentration exceeds the 6 wt.% solubility limit for in situ incorporation, surface deposition occurs. For the 10 wt.% sample, excess modifier particles block surface pores and partially obstruct water channels, explaining the conductivity reduction despite higher overall hydration capacity.

### 3.4. Thermogravimetric Analysis

**Figure 5:**
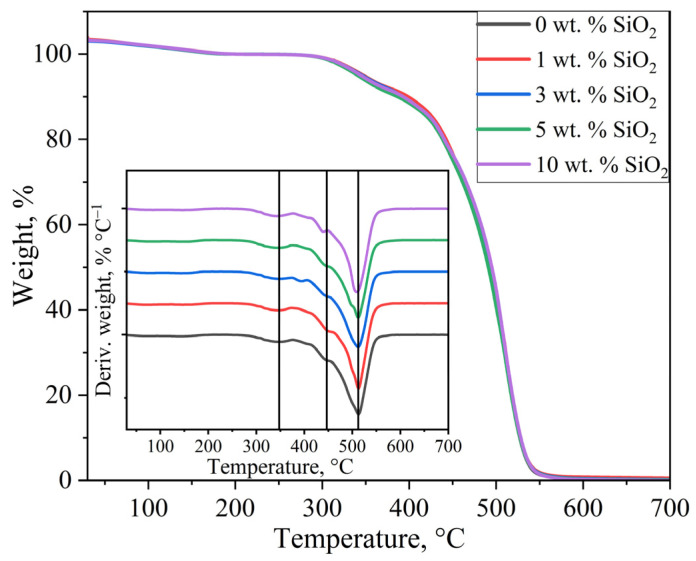
TGA and DTA curves of membrane samples.

Figure 5 shows the TGA curves for the investigated membrane samples. Initial mass loss below 200 °C was attributed to evaporation of residual water within the PEM. Consequently, the sample mass at 200 °C was taken as the reference value for subsequent analysis, leading to values above 100% on the TGA curves at lower temperatures. The similar profile of all TGA curves indicates that the modifier concentration has no significant effect on the thermal stability of the PEM matrix. Additionally, differential thermal analysis (DTA) was performed to determine the temperatures of maximum mass loss rate.

The DTA curves revealed distinct peaks associated with the degradation of various structural components of the PEM. In the temperature range of 340–350 °C, the observed peak corresponds to the decomposition of sulfonic acid groups, exhibiting a slight shift toward lower temperatures (from 348 °C for the unmodified membrane to 343 °C for the 10 wt.% SiO_2_-modified sample) with increasing modifier concentration. A characteristic peak at approximately 450 °C was attributed to the decomposition of side chains, which showed a notable 8 °C reduction only for the 10 wt.% SiO_2_ sample compared to the pristine membrane. The most prominent peak above 500 °C, corresponding to the degradation of the perfluorinated polymer backbone, remained unchanged in position across all samples, indicating that the incorporation of SiO_2_ nanoparticles does not affect the thermal stability of the hydrophobic polymer matrix. These results demonstrate that the introduction of SiO_2_ nanoparticles has only a minor influence on the thermal stability of PEMs at temperatures exceeding 300 °C. Importantly, within the critical operational temperature range of 30–300 °C for PEM fuel cells, all modified membranes maintained excellent thermal stability. The observed thermal behavior suggests that while SiO_2_ modification may slightly alter the decomposition kinetics of functional groups (sulfonic acid and side chains), it preserves the integrity of the primary polymer structure under typical fuel cell operating conditions.

The residual mass in the TGA curves was significantly lower than the measured modifier concentration, not exceeding 1 wt.% for all samples. This discrepancy results from the reaction of SiO_2_ with hydrogen fluoride, which forms during thermal decomposition of the ionomer backbone at around 300–500 °C. The degradation of SiO_2_ nanoparticles with the formation of gaseous products occurs via the following reaction [55,56]:

SiO_2_ + 4HF → SiF_4_ + 2H_2_O (23)

### 3.5. Electron Microscopy and Energy-Dispersive X-Ray Spectroscopy

**Figure 6:**
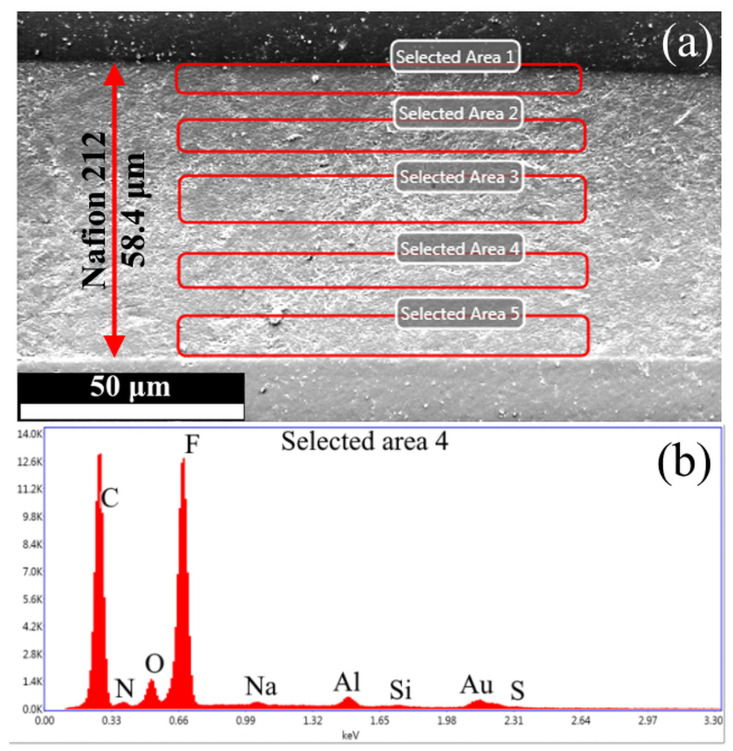
(**a**) An SEM micrograph of the PEM cross-section and (**b**) an EDS spectrum of the selected region.

Figure 6 presents (a) an SEM micrograph of the PEM cross-section and (b) an EDS spectrum of the selected region, respectively.

**Figure 7:**
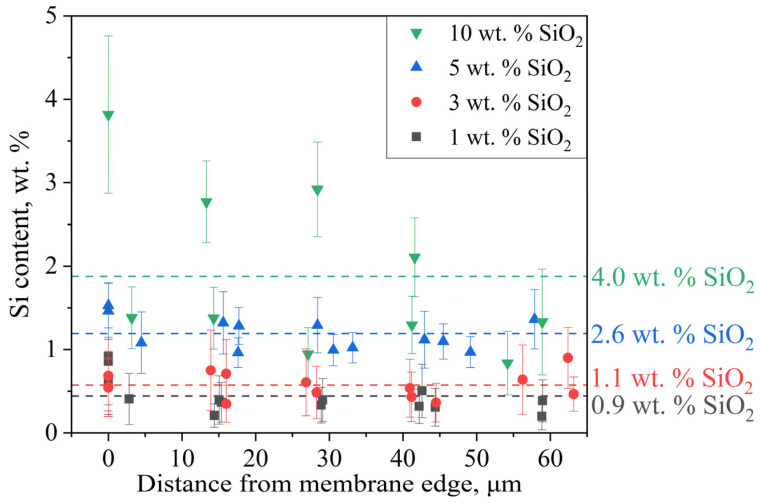
Distribution of the modifier in the membrane volume. Average values are indicated by the dotted line.

The SEM micrographs reveal an increase in membrane thickness from 50 μm to ~60 μm, likely due to solvent-induced swelling during epoxy resin embedding. The EDS spectrum shows characteristic peaks of Al (from microscope components) and Au (from sample coating), which were systematically excluded during Si concentration calculations. Figure 7 presents the quantified silicon concentration across analyzed membrane regions as a function of distance from the cross-section’s upper edge.

Due to the significantly lower mass of Si compared to the polymer matrix, the measured concentrations exhibit substantial experimental uncertainty. Consequently, in samples with low modifier content (1–3 wt.% SiO_2_), the EDS-derived concentrations approach the method’s detection limit, making meaningful quantification challenging. For higher-loading samples (5–10 wt.%), the spatial concentration trends remain discernible, though with notable edge enrichment—a consequence of preferential surface deposition during synthesis. This surface accumulation effect also explains the systematic discrepancy between the lower bulk SiO_2_ concentrations measured by EDS and the higher values obtained through gravimetric analysis. The 10 wt.% sample displayed particularly heterogeneous Si distribution, indicating modifier aggregation. Collectively, these results confirm successful in situ formation of SiO_2_ nanoparticles within the PEM bulk and their uniform dispersion at low concentrations (<5 wt.%), as required for homogeneous property enhancement.

**Figure 8:**
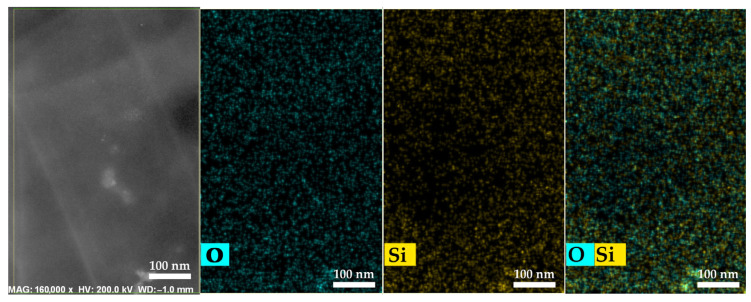
HAADF-STEM and elemental mapping images of Nafion^®^ 212 membranes containing 1 wt.% SiO_2_.

The micrograph and elemental mapping results confirm a high degree of distribution uniformity of SiO_2_ nanoparticles within the PEM bulk (Figure 8).

### 3.6. Small-Angle X-Ray Scattering

**Figure 9:**
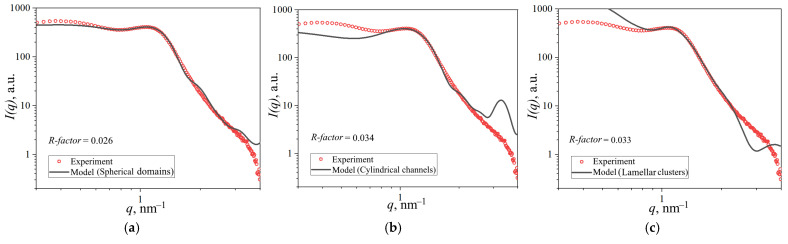
SAXS data for Nafion^®^ 212 membrane with theoretical fits corresponding to (**a**) spherical domain model, (**b**) cylindrical channel model, and (**c**) lamellar cluster model.

The internal membrane structure was investigated using SAXS. Given the lack of consensus in the literature regarding optimal structural models, we evaluated three prevalent approaches: spherical domains, cylindrical channels, and lamellar clusters. These models were first tested against the scattering profile of the unmodified reference membrane. The corresponding fitted curves are presented in Figure 9.

To quantify the agreement between experimental and theoretical scattering profiles, we calculated the *R-factor* within the ionic domain peak region (0.8–2.0 nm^−1^). All models showed satisfactory convergence with experimental data in this range, yielding *R-factors* of 0.026 (spherical), 0.034 (cylindrical), and 0.033 (lamellar). The spherical domain model demonstrated the best overall agreement across the full *q*-range. However, the cylindrical model exhibited a pronounced theoretical peak at *q* = 3 nm^−1^ that was substantially attenuated in experimental data, along with underestimated intensity at low *q*-values. This discrepancy could potentially be addressed by incorporating scattering contributions from lamellar structures in the hydrophobic matrix. The lamellar cluster model similarly showed deviations at both low and high *q*-values. Based on this systematic evaluation, we selected the spherical domain model for subsequent analysis. The parameters derived from calculations for each model are summarized in Table 1.

**Figure 10:**
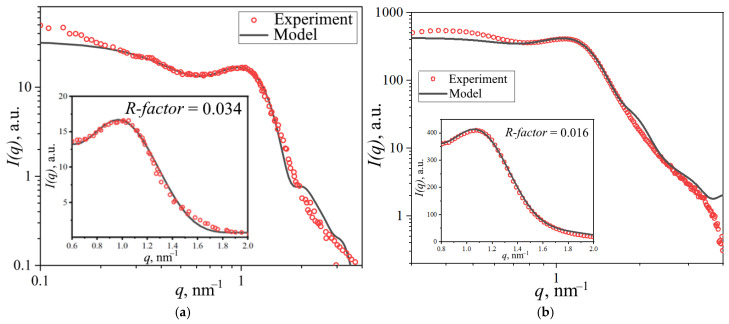
Experimental data fitting results from (**a**) reference [43] and (**a**,**b**) this work, showing theoretical curves corresponding to the spherical domain model.

The spherical domain model calculations were validated against reference data from [43], where an identical membrane type and structural model were employed. Figure 10 presents the fitted scattering profile for Nafion^®^ 212, demonstrating excellent agreement with both our experimental data and the literature results.

Table 2 compares the parameters obtained in this work with literature data from [43]. While most values show excellent agreement, minor discrepancies in *σ_R_* and *z*’, parameters likely arise from differences in optimization methodologies. Crucially, the mean channel diameters—key for evaluating structural transformation mechanisms—are identical between studies. For our PEM, the lower value of the water volume fraction *φ_w_* (attributed to reduced ion-exchange capacity from alternative membrane pretreatment) correlates with decreased aqueous cluster radius (*R_w_*) and increased intercluster distances (*D*, *αD*).

**Figure 11:**
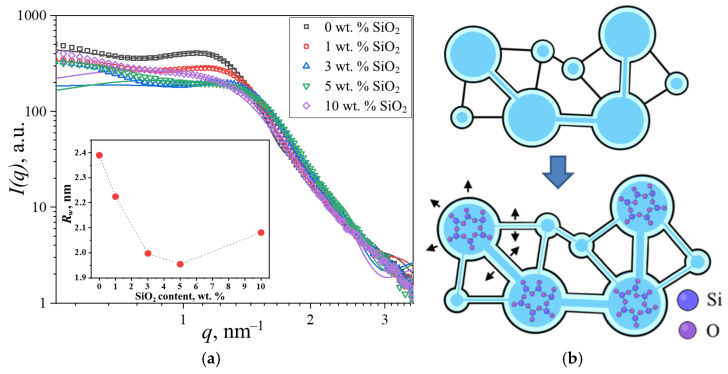
(**a**) Experimental and theoretical SAXS profiles of modified PEMs with inset showing mean domain radii (*R_w_*). (**b**) Proposed schematic of ionic domain structural evolution following SiO_2_ incorporation, the stretching of domain walls and channels is indicated by black arrows.

Further investigations focused on SiO_2_-modified membranes. Increasing the modifier content reduced the ionomer peak intensity due to diminished scattering contrast between the polymer and SiO_2_, which exhibit similar scattering length densities, as shown in Section 2.3. The processed SAXS profiles and corresponding spherical domain model fits are presented in Figure 11a.

The geometrical parameters of ionic domains in membranes with different SiO_2_ concentrations are presented in Table 3. With increasing modifier concentration up to 5 wt.% SiO_2_, the domain radius decreased from 2.39 nm to 1.96 nm, and interdomain distance decreased from 4.80 nm to 3.72 nm. Taking into account the increased water uptake of modified PEMs and the constrained elasticity model of water domain walls discussed in [18], a model of structural changes was proposed, as shown in Figure 11b. According to this model, water redistribution occurs from aqueous domains containing modifier nanoparticles into interdomain channels. This redistribution of water causes reduced mean interdomain distance (*D*) through the opening of closed channels and may enhance domain connectivity, resulting in improved specific conductivity of the membrane. Additionally, stretching of the modified domain walls takes place. Since SiO_2_ nanoparticles are more likely to form in larger ionic domains, and due to the similar scattering length density values of the modifier material and polymer compared to water, the analysis of SAXS data using the spherical domain model shows that the presence of large modifier-filled domains does not result in an increase in the average size of water domains.

**Figure 12:**
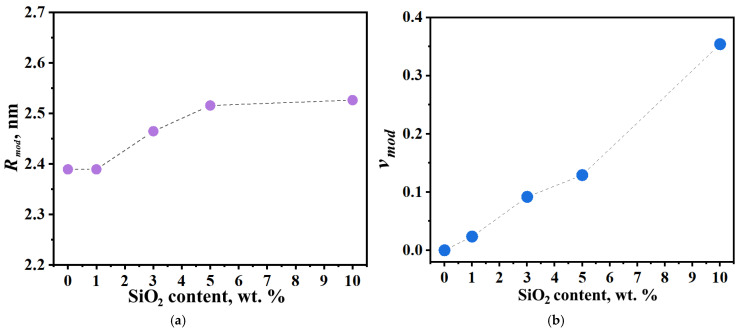
(**a**) Radius (*R_mod_*) and (**b**) volume fraction (*ν_mod_*) of modified clusters in PEM samples with varying SiO_2_ concentrations.

Incorporating modified domains into the model enables determination of both the average size of SiO_2_-containing domains (*R_mod_*) and their volume fraction within the membrane (*ν_mod_*). Figure 12 shows the dependence of these parameters on modifier concentration. The size of modified clusters increases progressively until reaching the wall stretchability limit at 10 wt.% modifier loading, with a maximum average domain diameter of 5 nm observed. Increasing modifier concentration also leads to growth in the volume fraction of the modified domain. For the 10 wt.% SiO_2_ sample, the combination of high modified domain fraction and maintained water uptake capacity causes expansion of the unmodified domain due to water displacement from modified to neighboring domains. However, at this high concentration, the blocking effect of the modifier outweighs the enhanced domain connectivity, resulting in an overall decrease in membrane conductivity.

## 4. Conclusions

The effects of sol–gel modification on Nafion membranes were investigated using TGA, electron microscopy, and SAXS. Three structural models of hydrated PEMs were evaluated: (1) core–shell spherical domains, (2) cylindrical channels, and (3) lamellar clusters. The spherical model demonstrated excellent agreement with experimental data across a wide *q*-range. The proposed structural transformation model explains water redistribution from SiO_2_-containing aqueous domains to interdomain channels, enhancing domain connectivity and proton conductivity. Quantitative analysis revealed that increasing modifier concentration up to 10 wt.% expands modified cluster size to 5 nm and their volume fraction. Beyond this threshold, the dominant blocking effect of excess SiO_2_ nanoparticles overcomes connectivity improvements, reducing overall conductivity. These findings highlight the critical need for precise modifier concentration optimization (5–10 wt.% range) to maximize membrane performance.

## Figures and Tables

**Table 1:** Structural parameters of water-swollen Nafion^®^ membrane (φ_w_ = 0.335) for the spherical, cylindrical, and lamellar models.

Spherical Domain Model	Cylindrical Channel Model	Lamellar Cluster Model
*R_w_* = 2.39 nm	*R_w_* = 1.69 nm	*d_w_* = 2.16 nm
*D* = 4.80 nm	*D* = 5.29 nm	*d_w_* + *d_p_* = 4.88 nm
*σ_R_* = 0.18	*σ_R_* = 0.20	Δ = 0.97 nm
*z’* = 4.1	*z’* = 2.4	*N_diff_* = 12
*α* = 1.18	*α* = 1.25	*N* = 14

**Table 2:** Comparison of structural parameters of the Nafion^®^ membrane for the spherical domain model.

Parameters	Parameters from Work [43]	The Parameters Obtained in This Study for the SAXS Curve from the [43]	Parameters Obtained for the SAXS Curve from This Study
*φ_w_*	0.386	0.386	0.335
*R_w_* (nm)	2.44	2.44	2.39
*D* (nm)	4.68	4.68	4.80
*σ_R_*	0.25	0.21	0.18
*z*’	4.71	4.77	4.1
*α*	1.16	1.16	1.18

**Table 3:** Structural parameters of unmodified domains in membranes with varying SiO_2_ content.

Parameters	SiO_2_ Content (wt.%)				
	0	1	3	5	10
*φ_w_*	0.335	0.344	0.370	0.394	0.409
*R_w_* (nm)	2.39	2.22	2.00	1.95	2.08
*D* (nm)	4.80	4.43	3.88	3.72	3.91
*σ_R_*	0.18	0.19	0.18	0.18	0.18
*R_mod_* (nm)	2.39	2.39	2.46	2.52	2.53
*ν_mod_*	0	0.02	0.09	0.13	0.35

## Data Availability

Dataset available on request from the corresponding author.

## Abbreviations

The following abbreviations are used in this manuscript:

PEM	proton exchange membrane
PEMFC	proton exchange membrane fuel cell
SAXS	small-angle X-ray scattering
TEOS	tetraethyl orthosilicate
TGA	thermogravimetric analysis
DTA	differential thermal analysis
STEM	scanning/transmission electron microscope
SEM	scanning electron microscope
HAADF	high-angle annular dark field
EDX	energy-dispersive X-ray
ETD	Everhardt-Thornley detector
